# First person – Weizhuo Wang

**DOI:** 10.1242/dmm.050459

**Published:** 2023-08-29

## Abstract

First Person is a series of interviews with the first authors of a selection of papers published in Disease Models & Mechanisms, helping researchers promote themselves alongside their papers. Weizhuo Wang is first author on ‘ Disrupting Hedgehog signaling in melanocytes by SUFU knockout leads to ocular melanocytosis and anterior segment malformation', published in DMM. Weizhuo is a PhD student in the lab of Ling Hou at Wenzhou Medical University, Wenzhou, China, investigating ocular pigment cell biology and associated disease.


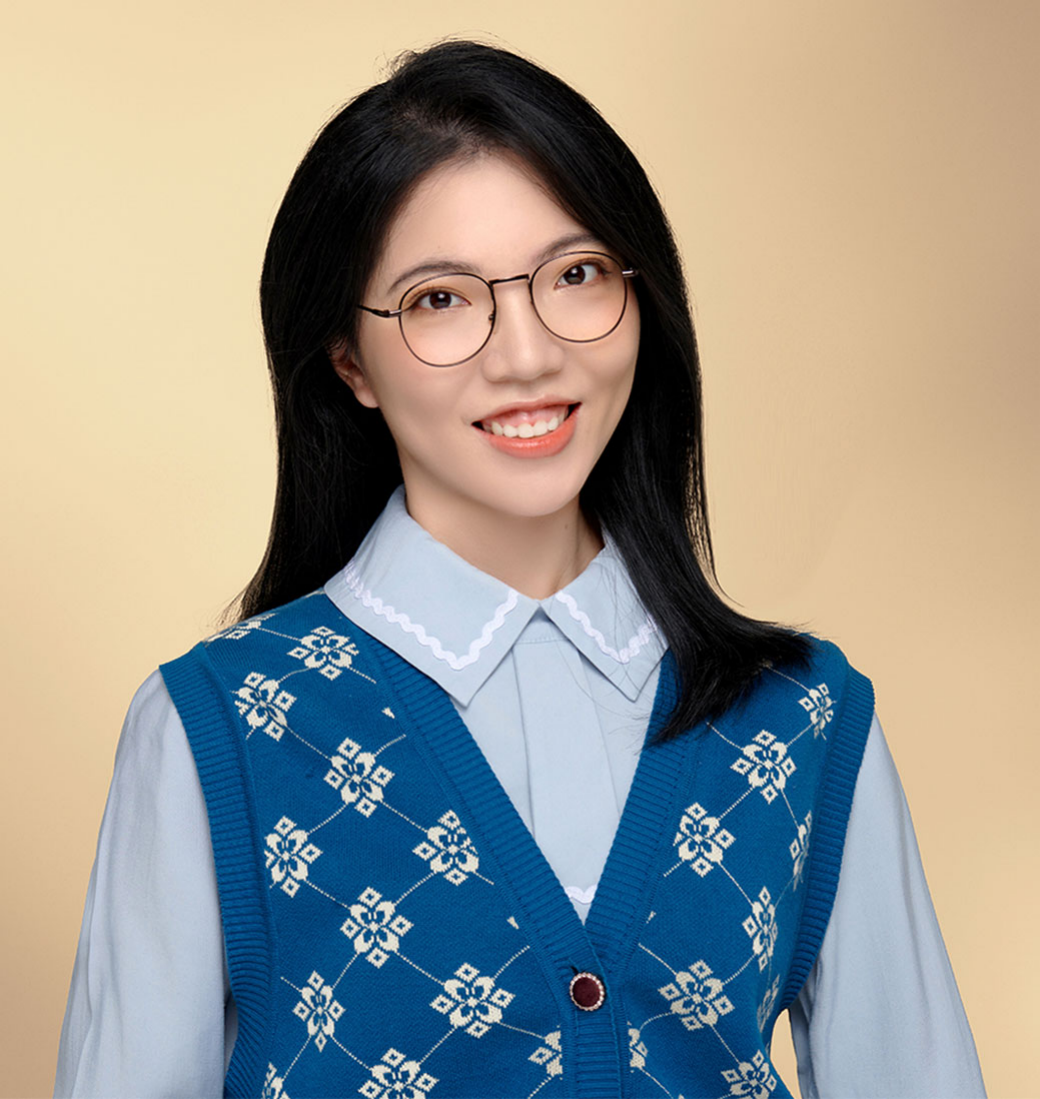


**Weizhuo Wang**

**How would you explain the main findings of your paper to non-scientific family and friends?**

We specifically disrupted the Hedgehog signaling regulator suppressor of fused (*Sufu*) in melanocytes. Interestingly, mice with conditional knockout (cKO) of *Sufu* (*Sufu*-cKO mice) are fully pigmented and show no developmental alterations in melanocyte number or distribution in skin and hair follicles. However, there are ectopic melanoblasts visible in the anterior chamber of the eye, which eventually displays severe malformation.

**What are the potential implications of these results for your field of research?**

This study identified that *Sufu* deficiency disrupts melanocytes selectively. Our results can help scientists and doctors gain insights into the pathogenesis of ocular anterior segment dysgeneses as seen in humans and shed light on potential therapeutic strategies.

**What are the main advantages and drawbacks of the experimental system you have used as it relates to the disease you are investigating?**

The Cre-LoxP recombination system allows us to specifically knock out *Sufu* in melanocytes, avoiding the early death of the cKO mutants in the embryonic period. It enables us to investigate the postnatal structural changes in mice. The drawback is that our results in the mouse model and in the melanocyte lineage still require validation in human samples.

[…] *Sufu* selectively disrupts melanocytes of the same origin.

**Figure:**
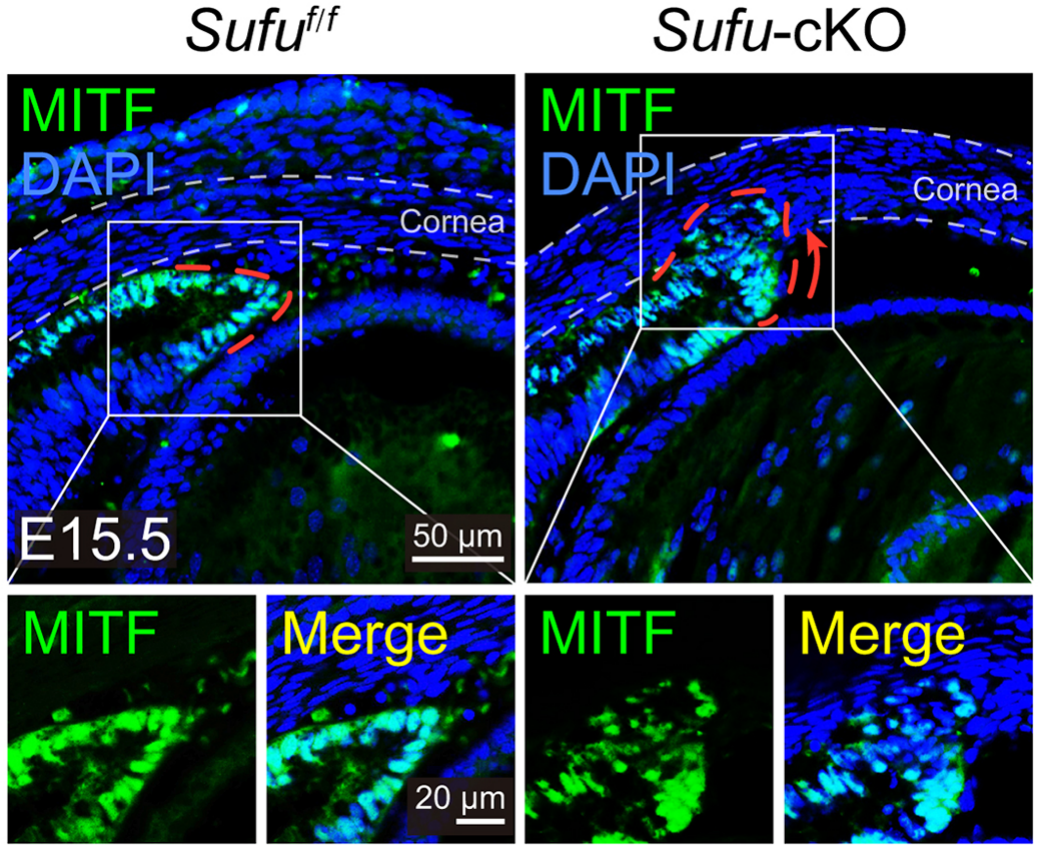
**Representative immunostaining images of MITF in embryonic day (E)15.5 *Sufu^f/f^* and *Sufu*-cKO eyes.** In contrast to control *Sufu^f/f^* eyes, *Sufu*-cKO eyes show ectopic MITF-positive melanoblasts (green) inserted into the cornea.

**What has surprised you the most while conducting your research?**

The most surprising result is that *Sufu* selectively disrupts melanocytes of the same origin. To be specific, *Sufu* deletion in melanocytes derived from neural crest cells leads to different changes in different organs: ocular melanocytes exhibit abnormal migration tendency, while cutaneous melanocytes are unaffected. Moreover, in the eye, only melanocytes located in the anterior segment migrate improperly during the period of development, whereas choroidal melanocytes do not change significantly. These findings not only contribute to the understanding of normal melanocyte homeostasis and the distinct roles of SUFU in subpopulations of ocular and cutaneous melanocytes, but may also help us understand the pathogenesis of melanocyte-related diseases in the human eye.

**What do you think is the most significant challenge impacting your research at this time and how will this be addressed over the next 10 years?**

The main challenge is to establish more suitable animal models for research. As is well known, the Cre-LoxP system has some off-target effects. In the future, more precise gene-editing systems will be developed and be used for scientific research.

[…] colleges and research centres need to provide more opportunities for young scientists to communicate and collaborate.

**What changes do you think could improve the professional lives of scientists?**

First, I think colleges and research centres need to provide more opportunities for young scientists to communicate and collaborate. For example, working with bioinformatic experts can make it possible to use current public databases more effectively and get more information. Second, it is important to create a relatively safe and relaxed environment for young researchers, so that they can be driven by their real intrinsic motivation.

**What's next for you?**

In the current study, we have demonstrated that *Sufu* is essential for maintaining melanocyte homeostasis. Next, I will continue to study the role of *Sufu* in ocular homeostasis and melanocyte-associated ocular diseases. In addition, I will be working as an ophthalmologist in a hospital. This will give me access to clinical sample resources, such as samples from patients with ocular melanocytosis, to further validate the results of the basic research.
